# Genetic relatedness and diversity of *Capillaria* species infecting bayad (*Bagrus bajad*) in upper Egypt

**DOI:** 10.1186/s12917-024-04076-x

**Published:** 2024-05-31

**Authors:** Salwa Mahmoud Abd-Elrahman, Salma M. Abdel-Rahman, Hanaa Y. Bakir, Ragaa A. Othman, Abeer A. Khedr, Mervat M. Khalifa, Sara S. Abdel-Hakeem

**Affiliations:** 1 Department of Parasitology, Faculty of Veterinary Medicine, Assiut University, Assiut, 71526 Egypt; 2 Department of Medical Parasitology Faculty of Medicine, Assiut University, Asyut, 71515 Egypt; 3 Department of Parasitology, Faculty of Veterinary Medicine, New Valley University, New Valley, El-Khargah, 72511 Egypt; 4 Parasitology Laboratory, Zoology and Entomology Department, Faculty of Science, Assiut University, Assiut, 71526 Egypt

**Keywords:** *Capillaria*, Fish, Genetic similarity, Relatedness, RAPD polymorphism

## Abstract

**Background:**

This study investigates the genetic characteristics of *Capillaria* isolates from the infected fish, *Bagrus bajad*, and their relation to human *Capillaria philippinensis* using Random Amplified Polymorphic DNA (RAPD-PCR) analysis. Fifteen fish *Capillaria* were isolated and compared to identified human *C. philippinensis* using six primers: M-are, M-1, G-7, G-11, G-15, and G-18.

**Results:**

All six primers successfully amplified DNA, highlighting their efficacy in distinguishing between human and fish *Capillaria* isolates. The analysis revealed distinctive banding patterns between fish and human isolates, with variations in size and number of DNA fragments. Additionally, genetic similarity analysis showed intriguing patterns of relatedness, with certain pairs exhibiting high similarity percentages. Comparative assessment of RAPD polymorphism demonstrated consistent findings of 100% polymorphism across all primers. The Unweighted Pair Group Method with Arithmetic Mean Algorithm (UPGMA) evaluated the closest relationship between human and fish isolates. These results underscore the utility of RAPD analysis in delineating the genetic diversity among *Capillaria* isolates from different hosts.

**Conclusion:**

Overall, this study contributes to our understanding of the genetic variability and relatedness among *Capillaria* isolates, shedding light on their evolutionary dynamics and zoonotic potential.

**Supplementary Information:**

The online version contains supplementary material available at 10.1186/s12917-024-04076-x.

## Introduction

*Capillaria* is a minute cylindrical worm predominantly caused by the nematode *C. philippinensis* [1]. Fish serve as intermediate hosts for the parasite, facilitating transmission to vertebrates, particularly humans, through the consumption of raw or undercooked fish containing infective larvae located within the muscles or internal organs [2]. Upon ingestion, the larvae of *C. philippinensis* by the vertebrate host penetrates the intestinal wall, where they develop into adult worms, initiating the onset of intestinal capillariasis [3]. It is recognized as an emerging and reemerging zoonotic disease, with its prevalence primarily concentrated in Southeast Asia, particularly in the Philippines, hence its nomenclature [4]. However, this parasite has also been documented in neighboring countries such as Thailand and Egypt [5]. Symptoms of the infection vary and may include chronic diarrhea, abdominal pain, malabsorption, weight loss, significant protein and nutrient depletion, and malnutrition [6]. Therefore, timely diagnosis of *C. philippinensis* infection is crucial due to its potential to cause severe, and sometimes fatal, outcomes when left untreated [7]. Various studies have employed polymerase chain reaction (PCR) for the accurate and sensitive detection of *C. philippinensis* DNA [8]. This molecular approach facilitates the identification of isolates through PCR and restriction fragment length polymorphism (RFLP), both recognized for their reliability, sensitivity, specificity, and reproducibility in characterizing polymorphic variants within the DNA sequence of the organism [9, 10].

Random Amplified Polymorphic DNA (RAPD) is a molecular technique that employs short, arbitrary primers to amplify random segments of DNA throughout the genome. This process generates distinct banding patterns, enabling differentiation between various strains or isolates of the parasite [11]. Furthermore, it offers a rapid and sensitive means of detecting the parasite’s DNA in clinical samples, such as stool or tissue specimens, even at low concentrations [12]. This sensitivity is particularly valuable for early detection and accurate diagnosis, particularly in scenarios where conventional methods may produce false negatives [13]. Additionally, RAPD analysis facilitates the genetic characterization and differentiation of *C. philippinensis* isolates. However, while RAPD presents numerous advantages for parasitic diagnosis, there is a scarcity of information regarding the application of RAPD for diagnosing *C. philippinensis* specifically. By comparing the RAPD profiles of diverse isolates, researchers can gain insights into the parasite’s genetic diversity and population structure, thereby informing epidemiological investigations and aiding in tracking infection spread [14].

Hence, the primary objective of this study is to evaluate the genetic diversity of *Capillaria* species isolates from the infected fish, *Bagrus bajad*, using RAPD analysis. Additionally, the study seeks to estimate the genetic relatedness between the *Capillaria* species in fish and *C. philippinensis* in human isolates. Such insights are crucial for comprehending the evolutionary connections and transmission patterns of the parasite. Furthermore, they aid in identifying potential reservoir hosts or sources of infection, thereby contributing to improved disease management strategies.

## Materials and methods

### Ethical declaration

This study adhered to both national and international ethical guidelines. Approval for the research was obtained from the Research Ethical Committee of the Faculty of Veterinary Medicine, Assiut University (Approval Number: 06/2024/0176).

### Parasite collection and preparation

A total of 50 bayed fish, *Bagrus bajad*, were collected from fishermen or markets and transferred directly to the Parasitology Department, Faculty of Medicine, Assiut University. Adult and larvae *Capillaria* were then extracted from the intestines of fifteen positive samples. Each fish sample was assigned to an identification code (F1- F15). Subsequently, the collected samples were divided into two separate tubes, washed with phosphate buffer saline (PBS), aliquoted, and stored at -80 °C for subsequent analysis. Similarly, human sample collection involved isolating adult worms from five freshly voided stool specimens obtained from the infected individuals. The human *C. philippinensis* was previously identified morphologically and molecularly using nested PCR, as described by Khalifa et al. [6]. . The human samples were collected specifically on the second day after administering katrex (levamisole) and were each assigned a distinct identification code (H1-H5) for tracking purposes.

### Genomic DNA extraction and RAPD-PCR amplification for the collected samples

Genomic DNA extraction from all worm samples was conducted using the GeneJET™ Genomic DNA Purification Kit (Thermo Scientific), following the manufacturer’s protocol. PCR reagents, comprising DNA polymerase enzyme, PCR buffer, magnesium chloride, and deoxynucleotide triphosphates (dNTPs), were employed for the RAPD PCR reaction. The PCR Master Mix utilized in this study was sourced from Promega Company (Madison, WI, USA). Six primer sequences were selected for the genetic identification of the collected samples. Primer (M-1), sequence published by Welsh and McClelland [15], Primer (M-are), sequence published by Vahidi et al. [16]. , and four primer G Kits, including G-7, G-11, G-15, and G-18 (Operon Technologies, Alameda, CA). Detailed primer sequences and their respective G + C content are shown in Table 1.

**Table 1:** Sequences and G + C Content of the used primers for RAPD-PCR Amplification of *Capillaria* samples

Primer	Sequence 5’ for 3’	G + C content
M- are	ATC TGG CAAC	50%
M-1	AGG TCA CTGA	50%
G-7	GAA CCT GCGG	70%
G-11	TGC CCG TCGT	70%
G-15	ACT GGG ACTC	60%
G-18	GGC TCA TCTC	60%

For each sample, the PCR master mix was prepared in a total volume of 25 µl, consisting of 12.5 µl Master Mix, 1 µl forward primer, 6.5 µl DNA-free water, and 5 µl template of genomic DNA from the original sample. The cycling profile commenced with an initial denaturation step of seven minutes at 94 °C, followed by 40 cycles of denaturation at 94 °C for 15 s, annealing at 60 °C for 15 s, and extension at 72 °C for 30 s. The final cycle included an extension time of 10 min at 72 °C. Each amplification run included a negative control comprising DNAase-free water and negative tissue.

### Visualization and analysis of PCR products

After amplification, 10 µl of each PCR product was subjected to electrophoresis in a 2.5% agarose gel and stained with Ethidium bromide using horizontal gel electrophoresis equipment (Compact M, Biometra, Germany). Visualization of each DNA fragment was achieved under UV illumination. Gel images were analyzed using the Gel Imager and Documentation System (Compact M, Biometra, Germany). Fragment sizes were determined by comparison with a 1Kb plus DNA ladder (Life Technologies, GibcoBRL) [17].

### Data analysis

The data analysis was performed using the Windows Statistical Package for Social Sciences (SPSS, v.16) to explore the frequency and existing associations. Cluster analysis was applied to provide instinctive similarity relationships among fish and human isolates of *Capillaria.* The dendrogram was based on Nei’s unbiased genetic distances and Li’s coefficient [18], employing the Unweighted Pair Group Method with Arithmetic Mean (UPGMA) algorithm [19]. The software POPGENE (version 1.31) was utilized for this analysis [20].

## Results

DNA analysis from the fish and humans infected with *Capillaria* spp. was done using the six primers: M-are, M-1, G-7, G-11, G-15, and G-18. All six primers were successfully produced by amplified DNA with reproducible banding patterns, exhibiting distinct variations between the fish and human *Capillaria* species. Variations in both the size and number of the amplified fragments were observed across the different primers (Table 2).

**Table 2:** Comparison of band patterns and DNA fragments size between human and fish isolates of *Capillaria* using the different primers

	Human Capillaria Isolate		Fish Capillaria Isolate	
Primer	Total number of bands	MW of amplified DNA fragments (bp)	Total number of bands	MW of amplified DNA fragments (bp)
M-are	9	1633 − 500	13	2338–391
M-1	9	1651 − 650	19	2900–358
G-7	8	1235 − 262	19	2482–262
G-11	12	1237 − 353	17	1606–291
G-15	12	1728 − 450	16	1728–250
G- 18	11	1880 − 526	17	2377–410

**Fig. 1:**
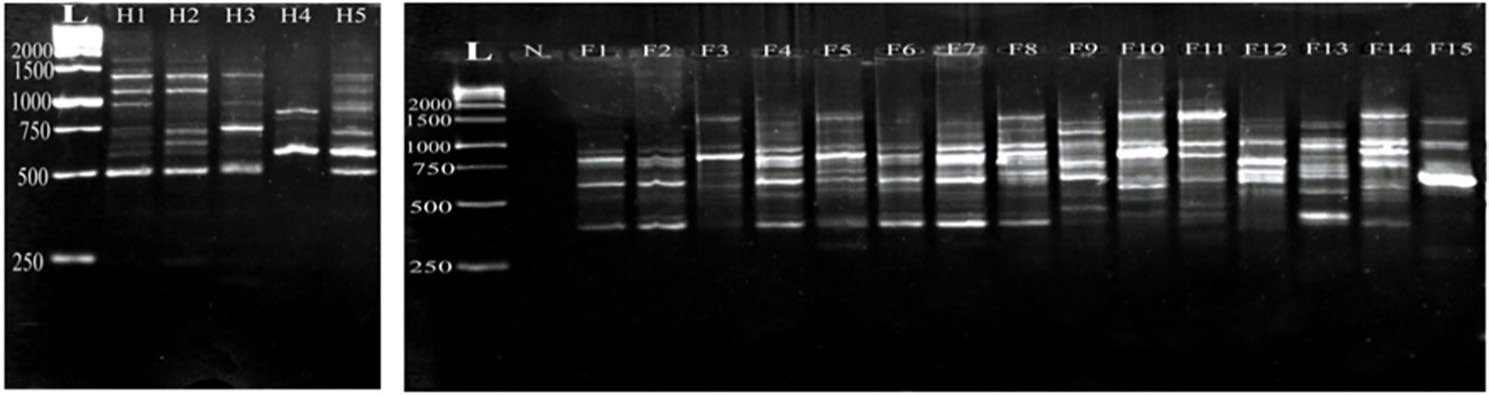
Comparative random amplified polymorphic DNA electrophoresis using M-are primer of *Capillaria* species isolated from *Bagrus bajad* (F1-F15) and human *C. philippinensis* (H1- H5). L: DNA ladder, N: negative control

Using the M-are primer, the fish *Capillaria* isolates exhibited different 13 amplified DNA fragments ranging from 2338 bp to 391 bp with a distinct polymorphic pattern. Fish samples (F1, F2, F6, and F7) have identical amplification patterns. Interestingly, the M-are primer demonstrated 92.3% polymorphism among all examined fish samples. In contrast, the human *C. philippinensis* displayed amplified DNA fragments ranging from 1633 bp to 500 bp, with varying intensity among the samples. Notably, two human samples exhibited identical amplification patterns (H1 and H2), while another sample (H4) showed fewer fragments at different molecular weights (600, 826, and 890 bp) with a polymorphism rate of 88.8% (Fig. 1).

**Fig. 2:**
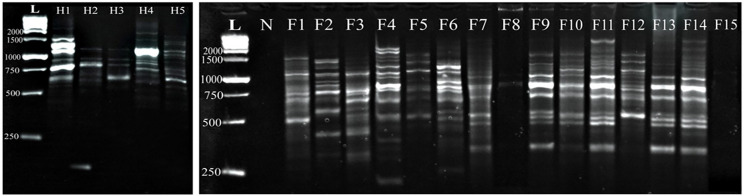
Comparative random amplified polymorphic DNA electrophoresis using M-1 primer of *Capillaria* species isolated from *Bagrus bajad* (F1-F15) and human *C. philippinensis* (H1- H5). L: DNA ladder, N: negative control

Similarly, the M-1 primer in the fish *Capillaria* isolates displayed 19 amplified DNA fragments ranging from 2900 bp to 358 bp, with unique polymorphic patterns observed. Notably, certain fragments were expressed in some specific fish samples. DNA fragments with molecular weights 1200, 1021, 867, 843, 740, 540, and 480 bp were expressed in F9, F10, F11, F13, and F14, while one sample (F15) failed to amplify with this primer. Amplified DNA fragments in the human *C. philippinensis* ranged from 1651 bp to 650 bp, with specific fragments (650–1130 bp), resulting in a 77.8% polymorphism rate (Fig. 2).

**Fig. 3:**
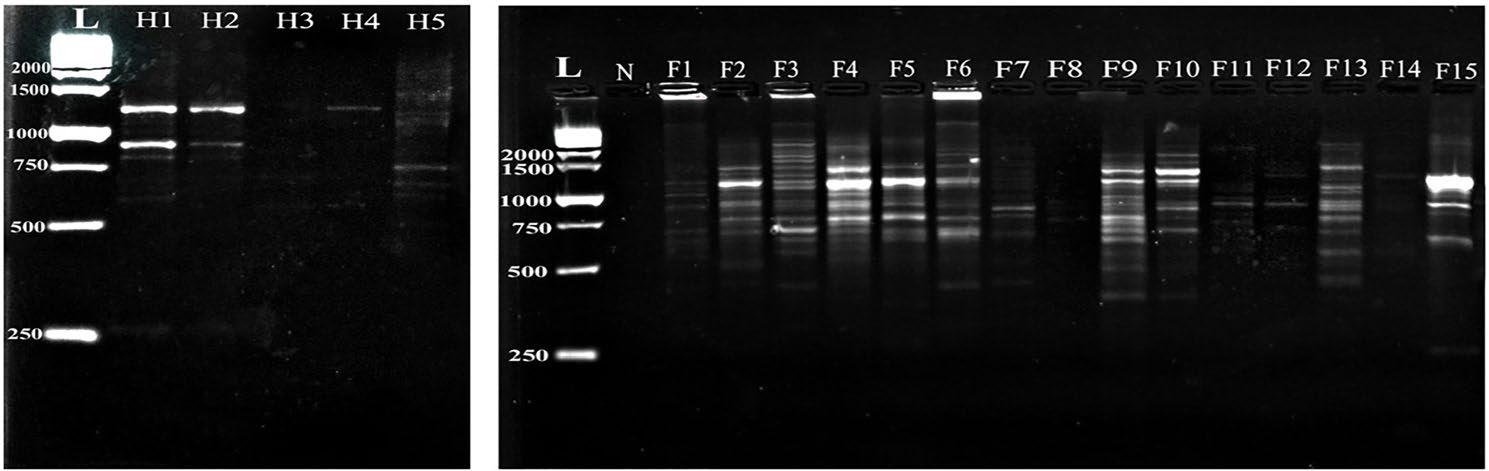
Comparative random amplified polymorphic DNA electrophoresis using G-7 primer of *Capillaria* species isolated from *Bagrus bajad* (F1-F15) and human *C. philippinensis* (H1- H5). L: DNA ladder, N: negative control

Moreover, the G-7 primer demonstrated that amplified DNA fragments in the fish *Capillaria* isolates exhibited 19 amplified fragments with distinct polymorphic patterns, leading to 100% polymorphism among all infected fish samples. The amplified DNA fragments of the human *C. philippinensis* ranged from 1235 bp to 262 bp, with varying expression levels among the infected samples. Notably, this primer showed 100% polymorphism among the human samples (Fig. 3).

**Fig. 4:**
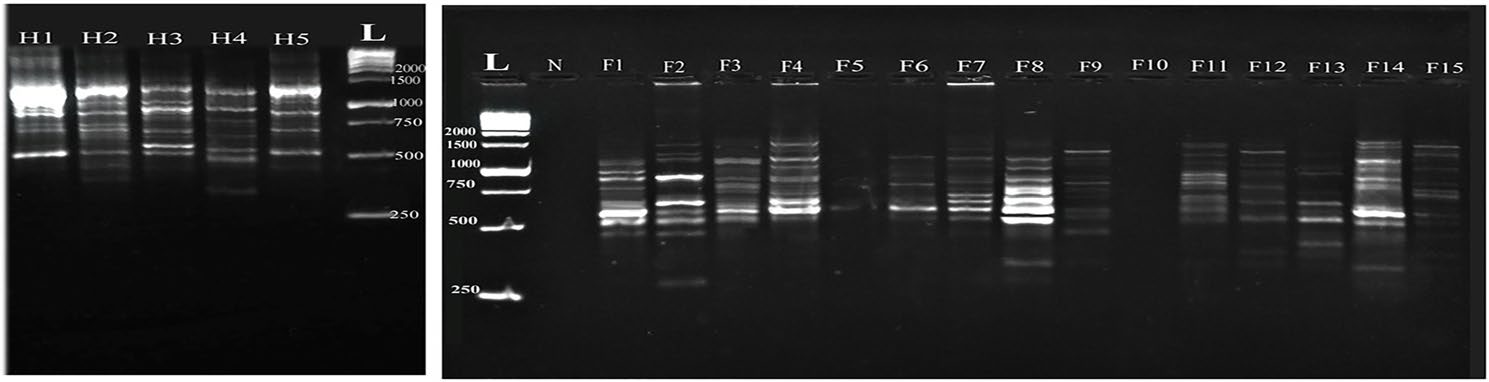
Comparative random amplified polymorphic DNA electrophoresis using G-11 primer of *Capillaria* species isolated from *Bagrus bajad* (F1-F15) and human *C. philippinensis* (H1- H5). L: DNA ladder, N: negative control

DNA amplification using the G-11 primer revealed that the fish *Capillaria* isolates exhibited 17 amplified fragments, albeit with distinct polymorphic patterns, and one sample failed to amplify (F10), resulting in 100% polymorphism among all examined fish samples. The human *C. philippinensis* showed 12 amplified DNA fragments ranging from 1237 bp to 353 bp, with identical patterns observed across all examined samples (Fig. 4).

**Fig. 5:**
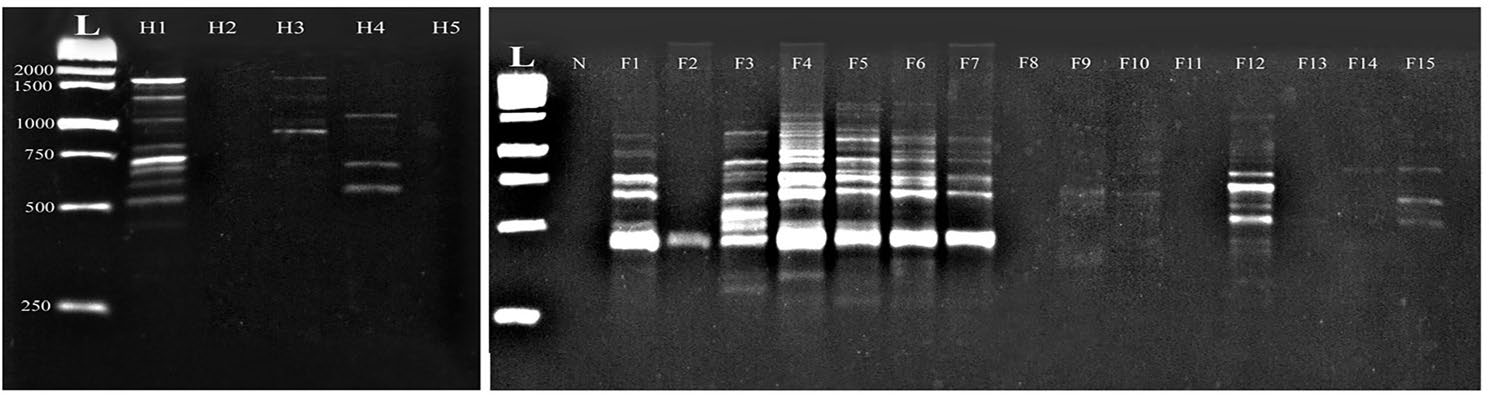
Comparative random amplified polymorphic DNA electrophoresis using G-15 primer of *Capillaria* species isolated from *Bagrus bajad* (F1-F15) and human *C. philippinensis* (H1- H5). L: DNA ladder, N: negative control

The G-15 primer in the fish *Capillaria* isolates exhibited 16 amplified fragments with prominent molecular weights of 1728 bp and 250 bp, which is similar to the human *C. philippinensis* isolates. Whereas specific fish samples failed to amplify (F8 and F11), resulting in a 100% polymorphism rate. The human *C. philippinensis* isolates displayed 12 amplified DNA fragments ranging from 1728 bp to 450 bp, whereas two human samples (H2 and H5) failed to amplify DNA with the G-15 primer (Fig. 5).

**Fig. 6:**
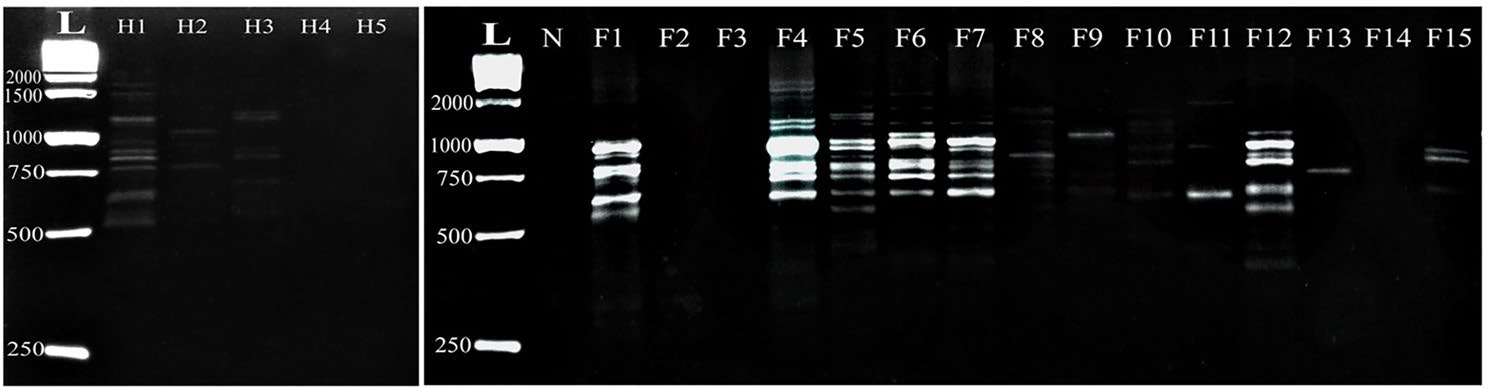
Comparative random amplified polymorphic DNA electrophoresis using G-7 primer of *Capillaria* species isolated from *Bagrus bajad* (F1-F15) and human *C. philippinensis* (H1- H5). L: DNA ladder, N: negative control

The G-18 primer in the fish *Capillaria* isolates showed 17 amplified fragments, with specific molecular weights being more prominent at 2377 and 410 bp. Notably, certain fish samples (F2, F3, and F14) were not amplified by the G-18 primer, leading to a 100% polymorphism rate. The human *C. philippinensis* isolates exhibited 11 weak and faintly amplified DNA fragments ranging from 1880 bp to 526 bp. The human samples H4 and H5 failed to amplify with the G-18 primer, resulting in a 100% polymorphism rate among the examined samples (Fig. 6).

### Analysis of DNA polymorphism between the fish *Capillaria* isolates

DNA polymorphism was examined among the fish *Capillaria* isolates using the same set of primers, resulting in the amplification of 101 fragments. The fragment sizes ranged from 262 bp (G-7 primer) to 2901 bp (M-1 primer). Most primers exhibited a 100% polymorphism rate, except the M-are primer which showed a polymorphism rate of 92.3% (Table 3).

**Table 3:** RAPD polymorphism among fish samples

Primer	Total number of bands (a)	Amplified fragment size range (bp)	Number of polymorphic bands (b)	Polymorphism (b/a) %
M-are	13	2338 − 391	12	92.3
M-1	19	2900–358	19	100
G-7	19	2482 − 262	19	100
**G-11**	17	1606 − 291	17	100
**G-15**	16	1728 − 288	16	100
**G- 18**	17	2377 − 410	17	100
**Total**	101	2901 − 262	100	99

### Analysis of DNA polymorphism between the human *C. philippinensis* isolates

DNA polymorphism was observed among the human *C. philippinensis* isolates, resulting in the amplification of 59 fragments. Of these, 53 fragments exhibited polymorphism, with a polymorphic rate of approximately 89.8%. The amplified DNA fragments varied in size, ranging from 262 bp with the primer G-7 to 1880 bp with the primer G-18. Notably, three primers (G-7, G-15, and G-18) displayed a 100% polymorphism rate, whereas the G-11, M-1, and M-are primers showed varying rates of polymorphism at 66.6%, 77.8%, and 88.8%, respectively (Table 4).

**Table 4:** RAPD polymorphism among human samples

Primer	Total number of bands (a)	Amplified fragment size range (bp)	Number of polymorphic bands (b)	Polymorphism (b/a) %
M-are	9	1633 − 500	8	88.8
M-1	9	1651 − 650	7	77.8
G-7	8	1235 − 262	8	100
G-11	12	1237 − 353	8	66.6
G-15	11	1728 − 450	12	100
G- 18	10	1880 − 526	10	100
Total	59	1880 − 262	53	89.8

### Genetic polymorphism and relatedness between the fish and human *Capillaria* isolates

RAPD polymorphism was assessed among both fish *Capillaria* isolates and human *C. philippinensis*. A total of 101 fragments were amplified, ranging in size from 262 bp with the G-7 primer to 2901 bp with M-1 primer, displaying 100% polymorphism. The details of the used primers and the number of amplified DNA fragments are presented in Table 5.

**Table 5:** RAPD polymorphism among human and fish *Capillaria* isolates

Primer	Total number of bands (a)	Amplified fragment size range (bp)	Number of polymorphic bands (b)	Polymorphism b/a %
M-are	13	2338 − 391	13	100
M-1	19	2900–358	19	100
G-7	19	2482 − 262	19	100
G-11	17	1606 − 291	17	100
G-15	16	1728 − 288	16	100
G- 18	17	2377 − 410	17	100
Total	101	2901 − 262	101	100

### Genetic similarity among human and fish *Capillaria* isolates

The highest genetic similarity percentage (68.5%) among fish *Capillaria* isolates was observed between the fish samples F5 and F6, followed by other pairs with slightly lower similarity percentages (Table 6). Whereas human *C. philippinensis* isolates exhibited various degrees of similarity between the different samples, with a highest similarity percentage (63.2%) between the H1 and H2 (Table 7).

**Table 6:** Genetic similarity percentage among fish *Capillaria spp*. samples

	F1	F2	F3	F4	F5	F6	F7	F8	F9	F10	F11	F12	F13	F14	F15
F1	-														
F2	58	-													
F3	50	56.7	-												
F4	67.4	48.2	55.6	-											
F5	61.3	42.4	54.8	62.9	-										
F6	60.5	53.7	59.5	64.4	68.5	-									
F7	61.3	51.5	49.3	67.4	66.7	68.5	-								
F8	41.4	49	39.3	33.3	36.4	35.7	43.6	-							
F9	41.1	40.6	45.1	43.7	42.9	42.3	48.6	34	-						
F10	41.3	33.3	45.9	36.4	50	49.2	40	18.6	48.3	-					
F11	49.3	53.3	41.8	45.8	36.4	47.8	51.5	36.7	46.9	51.9	-				
F12	58.7	33.3	46.6	53.9	52.8	57.5	58.3	29.1	34.3	43.3	36.4	-			
F13	35.8	41.4	49.2	42	34.4	46.2	50	29.8	51.6	38.5	55.2	37.5	-		
F14	37.7	40	38.8	36.1	21.2	29.9	36.4	36.7	46.9	44.4	66.7	30.3	48.3	-	
F15	32.3	26.4	40	34.2	27.1	26.7	33.9	28.6	35.1	21.3	41.5	44.1	35.3	30.2	-

**Table 7:** Genetic similarity percentage among human *capillaria spp*. samples

	H1	H2	H3	H4	H5
H1	-				
H2	63.2	-			
H3	46.4	39	-		
H4	34.6	37.8	27.8	-	
H5	45.2	51.1	43.5	42.9	-

### Phylogenetic relationship between human and fish *Capillaria* isolates

**Fig. 7:**
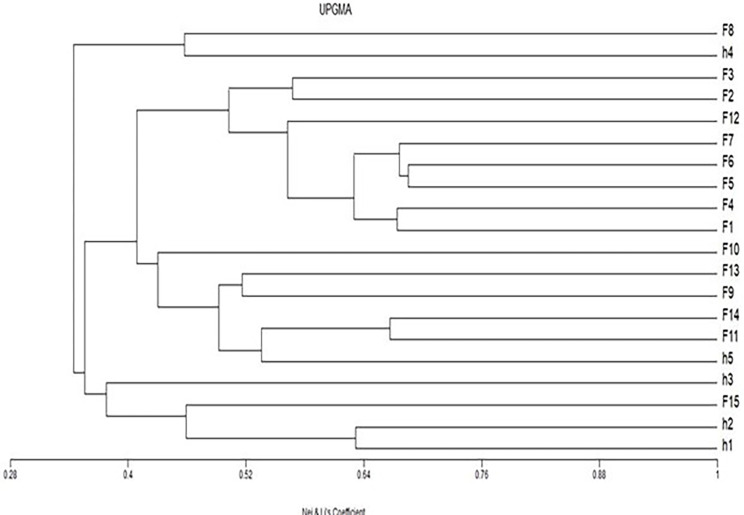
A dendrogram using the Unweighted Pair Group Method with Arithmetic Mean (UPGMA) algorithm showing the Nei and Li genetic dissimilarity coefficient matrix between *Capillaria* species isolated from *Bagrus bajad* (F1-F15) and human *C. philippinensis* (H1- H5)

Cluster analysis was conducted utilizing the Nei and Li genetic dissimilarity coefficient matrix, employing the UPGMA method, where the similarity between the different isolates of *Capillaria* can be observed (Fig. 7). A dendrogram was constructed with all the amplification bands obtained by RAPD using the six primers. Twenty groups were detected. Fish isolates (F5-F6) were grouped with the greatest similarity percentage (0.685), followed by F5, F6, and F7 (0.676). The nearest relationship between human and fish *Capillaria* isolates was observed between H5 and F11- F14 (0.536), followed by H5 and F9 - F13 (0.492), and between human isolate H4 and fish isolate F8 (0.46(.

## Discussion

Capillariasis, a significant zoonotic parasitic infection, poses a substantial threat to human health. The current study provides valuable insights into the zoonotic potential of these parasites and the degree of genetic relatedness between the fish *Capillaria.* spp. and human *C. philippiensis* isolates using RAPD-PCR. Previous research used RAPD markers to assess genetic variation between *Ascaridia galli* and *A. columbae* and found a high percentage (83%) of polymorphism [21]. In another report, RAPD and ISSR markers were used to study the genetic variability in *Capsicum* species, revealing genetic similarities and groupings [22]. These studies collectively highlight the utility of RAPD analysis in assessing genetic diversity and similarity in various organisms. According to the available scientific literature, the genetic characterization of *C. philippinensis* or fish *Capillaria* species is notably scarce. Hence, the present study represents one of the earliest attempts to explore the genetic variability and relatedness between human *C. philippinensis* and fish *Capillaria* spp. In selecting the six random primers utilized in our study, we referenced existing literature, specifically focusing on their documented efficacy in amplifying species belonging to the genus *Trichinella* within the superfamily Trichuroidea, which also encompasses the genus *Capillaria* [23]. This approach ensured the relevance and suitability of the selected primers for our research objectives.

Our findings revealed distinct banding patterns and variations in the size of DNA fragments across the tested isolates, indicating underlying genetic differences between them. The primers employed in this study for the amplification of *Trichocephalus trichuris* were previously documented [24]. Notably, distinct polymorphic patterns were observed using primers M-1, M-are, G-7, and G-18. Martinez et al. [25]. corroborated the efficacy of these primers by utilizing them for amplifying DNA extracted from *Trichuris trichiura* eggs obtained from infected patients.

When examining the band patterns and fragment size using the different primers, notable differences were observed between the fish *Capillaria* isolates and human *C. philippinensis*. Despite the use of the same set of primers, variations in the number and size of amplified fragments were evident, which reflected the genetic diversity within and between the two host species. For instance, primer M-are showed distinct banding patterns in both human and fish isolates, with varying polymorphism rates. The human *C. philippinensis* isolates displayed a range of fragment sizes, while the fish *Capillaria* isolates exhibited a different set of amplified fragments, indicating the genetic divergence between the two groups. Similarly, the M-1 primer produced contrasting banding patterns, highlighting the genetic differences between human and fish *Capillaria* isolates [26].

All six primers consistently produced amplified DNA with reproducible banding patterns, demonstrating a success rate of 100%. Interestingly, a study conducted by Osman et al. [27]. showed contrasting findings from our results. In their research, they utilized the same set of six primers for the amplification and characterization of adult *Capillaria* spp. collected from stomach of freshwater fish, specifically *Bagrus docmac* and *B. bayad* from Menoufia and Kalyobia Governorates, Egypt. However, in their study, all primers except the G-11 primer failed to amplify *Capillaria* spp., indicating a discrepancy between their outcomes and our results. Furthermore, the analysis using the primers G-7, G-11, G-15, G-18, and G-15 revealed consistent polymorphism rates within both human and fish isolates, underscoring the genetic heterogeneity within *Capillaria* populations [28].

The assessment of genetic similarity among human and fish *Capillaria* species further elucidated the evolutionary relationships between the different samples. The genetic similarity percentages revealed varying degrees of relatedness among isolates within each host species [29]. Interestingly, certain fish isolates exhibited a closer genetic similarity to human *C. philippinensis* isolates, suggesting potential cross-species transmission or shared evolutionary history [30]. Phylogenetic analysis using UPGMA clustering provided additional insights into the genetic relationships between fish *Capillaria* isolates and human *C. philippinensis*. The clustering patterns depicted in the dendrogram highlighted distinct genetic clusters and subgroups, further supporting the notion of evolutionary divergence and the potential host-specific adaptations within *Capillaria* populations [31].

Our findings can gain insights into the transmission dynamics of the parasite. This understanding is crucial for implementing targeted control measures to prevent transmission and reduce human infection rates [32]. Additionally, it can help identify potential reservoir hosts involved in the transmission cycle. This information aids in designing comprehensive control strategies targeting both reservoir hosts and intermediate hosts to interrupt the parasite’s life cycle and enhance the accuracy and efficiency of disease diagnosis, facilitating prompt treatment and control measures.

Future research avenues may involve further exploration of the evolutionary dynamics and transmission pathways of *Capillaria*, which could have implications for disease management and control strategies.

## Conclusion

In conclusion, our study provides comprehensive insights into the genetic diversity and relatedness of *Capillaria* isolates from fish and human hosts. Through RAPD-PCR analysis, we successfully characterized the DNA profiles of *Capillaria* isolates isolated from *Bagrus bajad* and human *C. philippinensis* using six primers. The results showed distinct banding patterns and polymorphic variations between the fish and human samples. The observed variations in the size and number of DNA fragments underscore the genetic heterogeneity among *Capillaria* isolates from different hosts. Furthermore, all primers exhibited high efficiency in amplifying DNA, highlighting their potential for discriminating against human and fish *Capillaria* strains. Our findings also shed light on the polymorphism rates among *Capillaria* isolates, with certain primers demonstrating higher polymorphism levels than others. Moreover, the genetic similarity analysis provided valuable insights into the relatedness among human and fish *Capillaria* isolates, suggesting potential evolutionary relationships between them. The consistent observation of 100% polymorphism across all primers in both human and fish isolates underscores the robustness of RAPD analysis in delineating genetic diversity in *Capillaria* species.

### Electronic supplementary material

Below is the link to the electronic supplementary material.

Supplementary Material 1

Supplementary Material 2

Supplementary Material 3

Supplementary Material 4

Supplementary Material 5

Supplementary Material 6

## Data Availability

No datasets were generated or analysed during the current study.
