# Supplementary figures and images for: Genetic relatedness and diversity of Capillaria species infecting bayad (Bagrus bajad) in upper Egypt
Source: BMC Vet Res. 2024 May 31;20:235. doi: 10.1186/s12917-024-04076-x (PMC11141003; doi:10.1186/s12917-024-04076-x)

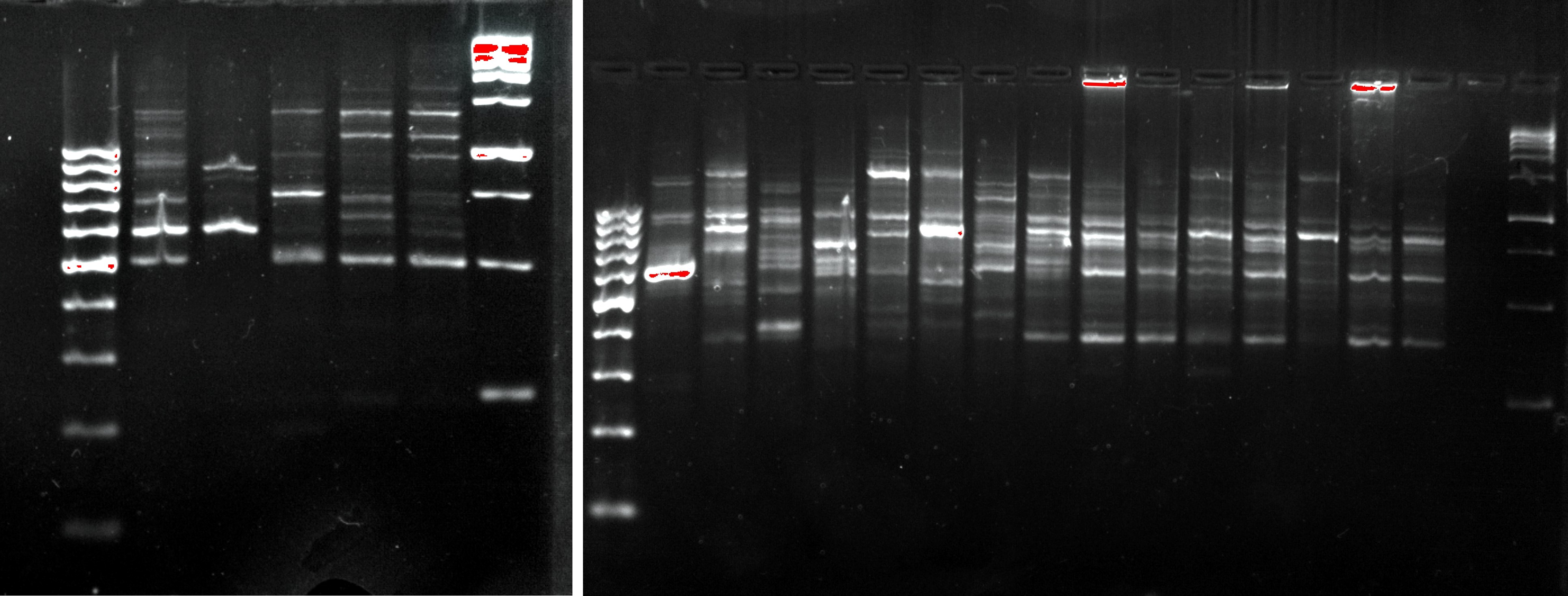

Supplement: Supplementary file 1 — Supplementary Material 1 [file 12917_2024_4076_MOESM1_ESM.jpg]

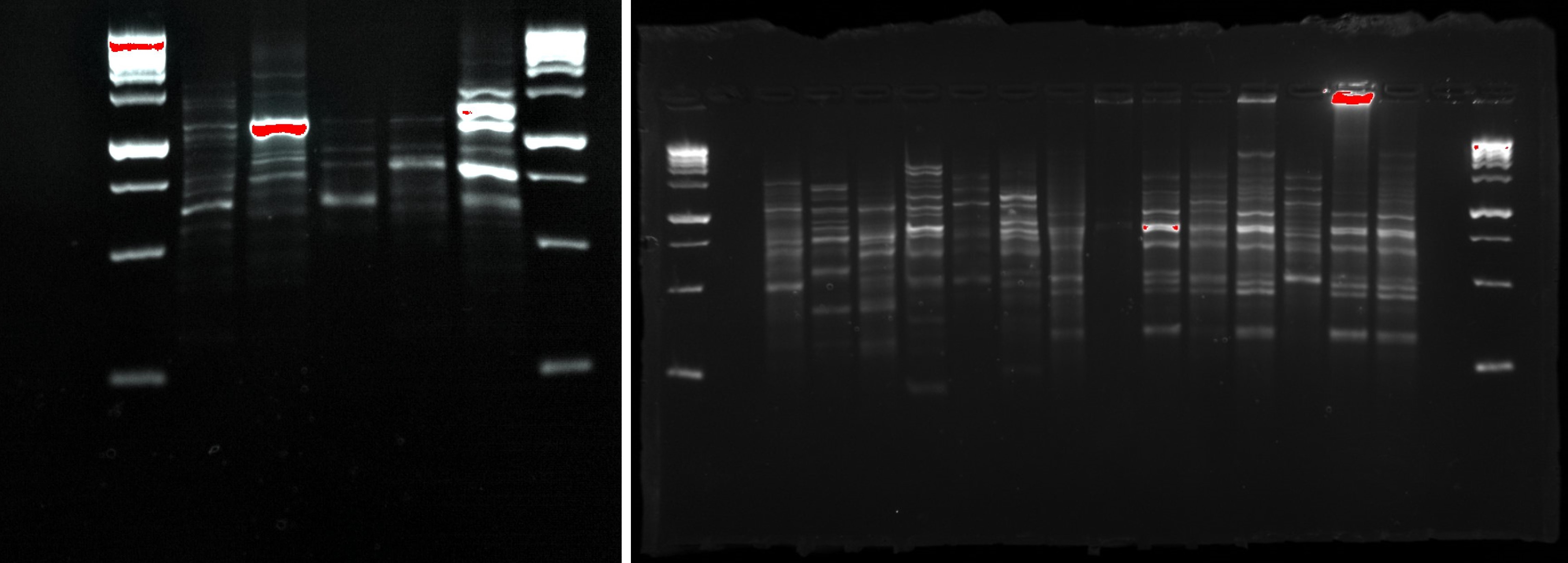

Supplement: Supplementary file 2 — Supplementary Material 2 [file 12917_2024_4076_MOESM2_ESM.jpg]

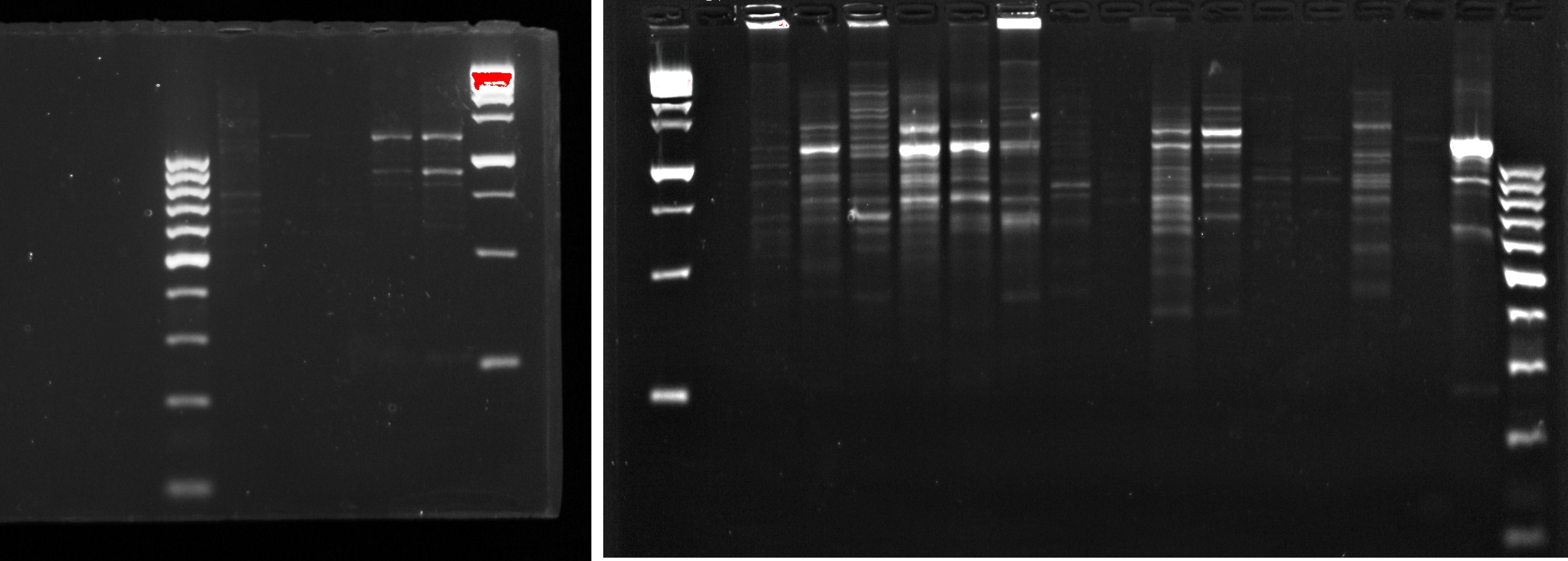

Supplement: Supplementary file 3 — Supplementary Material 3 [file 12917_2024_4076_MOESM3_ESM.jpg]

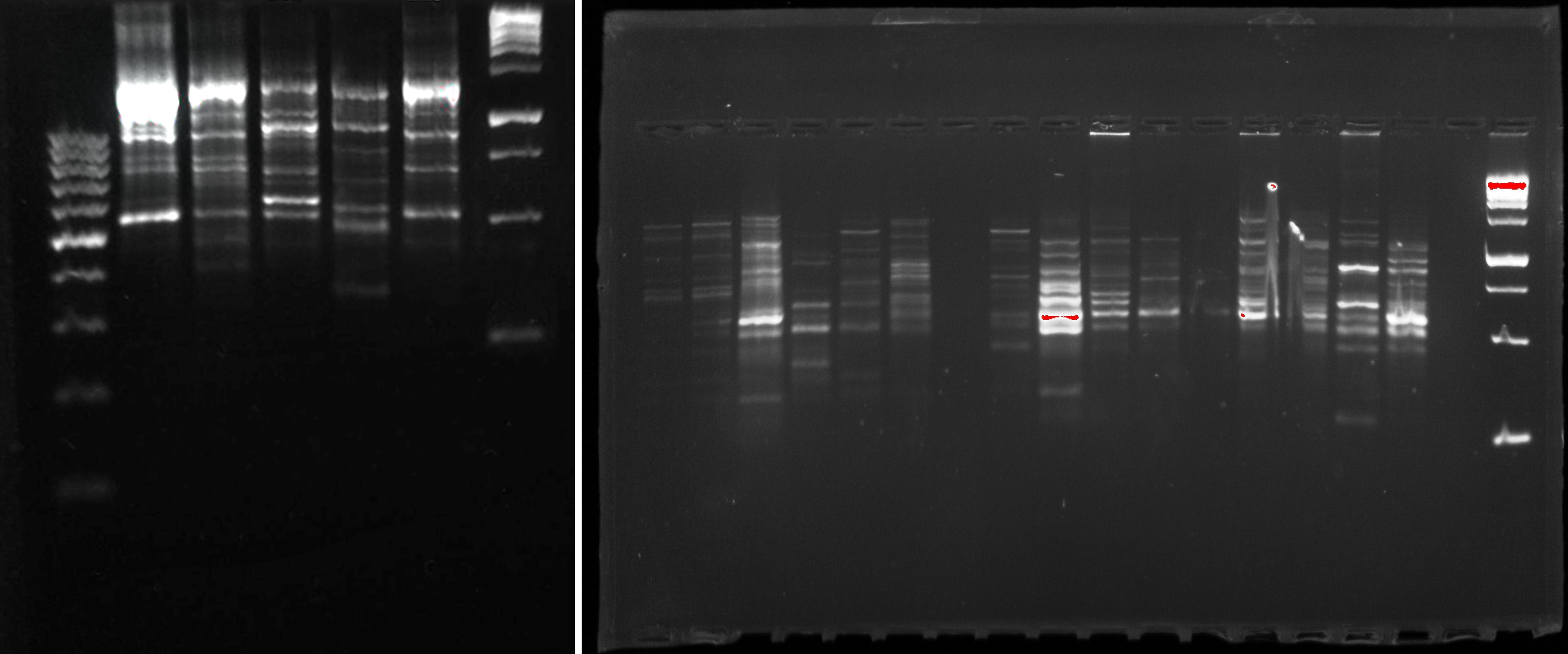

Supplement: Supplementary file 4 — Supplementary Material 4 [file 12917_2024_4076_MOESM4_ESM.jpg]

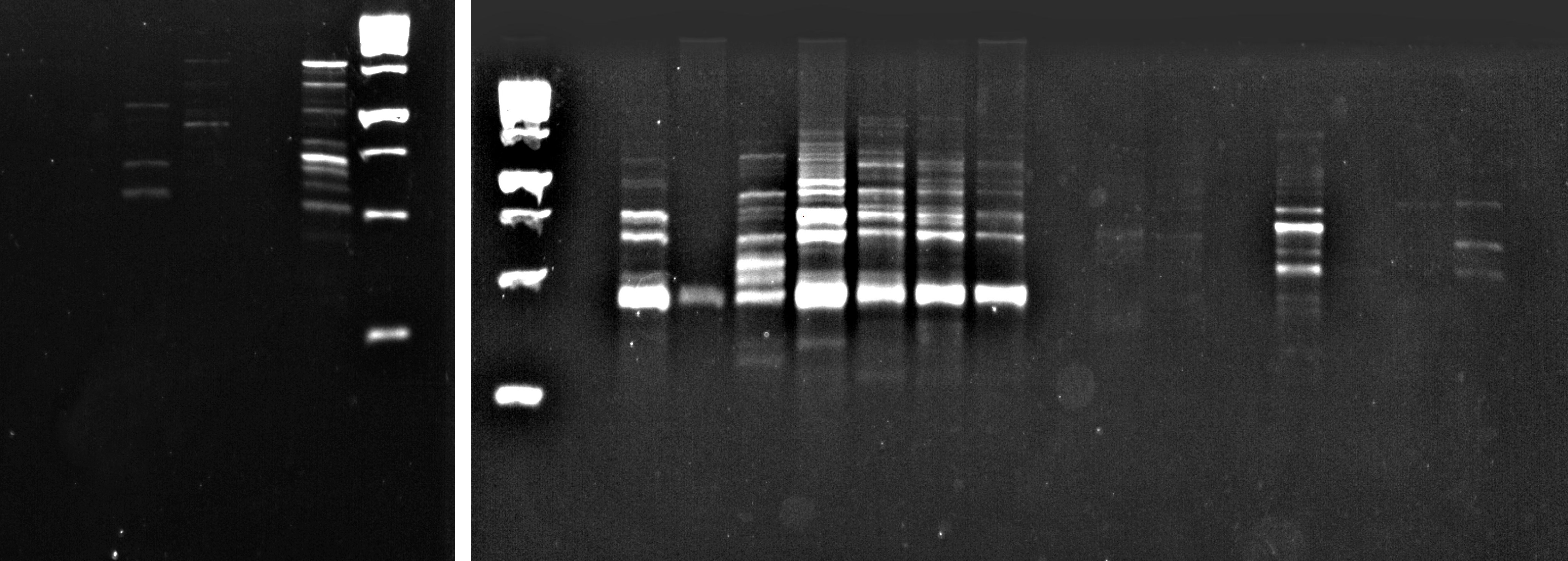

Supplement: Supplementary file 5 — Supplementary Material 5 [file 12917_2024_4076_MOESM5_ESM.jpg]

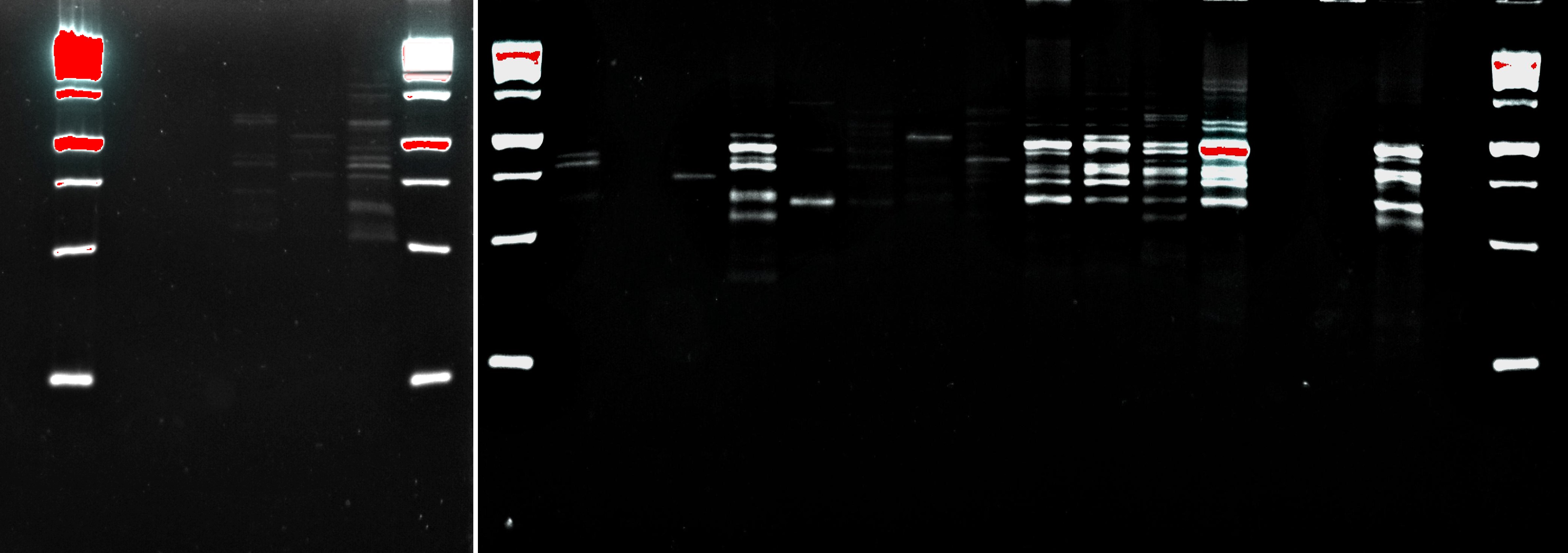

Supplement: Supplementary file 6 — Supplementary Material 6 [file 12917_2024_4076_MOESM6_ESM.jpg]
